# The Era of Thromboinflammation: Platelets Are Dynamic Sensors and Effector Cells During Infectious Diseases

**DOI:** 10.3389/fimmu.2019.02204

**Published:** 2019-09-13

**Authors:** Li Guo, Matthew T. Rondina

**Affiliations:** ^1^University of Utah Molecular Medicine Program, Salt Lake City, UT, United States; ^2^Department of Internal Medicine, University of Utah, Salt Lake City, UT, United States; ^3^Department of Pathology, University of Utah, Salt Lake City, UT, United States; ^4^George E. Wahlen VAMC Department of Internal Medicine and GRECC, Salt Lake City, UT, United States

**Keywords:** platelet, sepsis, inflammation, infection, dengue, malaria, bacteria, thromboinflammation

## Abstract

Platelets are anucleate cells produced by megakaryocytes. In recent years, a robust body of literature supports the evolving role of platelets as key sentinel and effector cells in infectious diseases, especially critical in bridging hemostatic, inflammatory, and immune continuums. Upon intravascular pathogen invasion, platelets can directly sense viral, parasitic, and bacterial infections through pattern recognition receptors and integrin receptors or pathogen: immunoglobulin complexes through Fc and complement receptors—although our understanding of these interactions remains incomplete. Constantly scanning for areas of injury or inflammation as they circulate in the vasculature, platelets also indirectly respond to pathogen invasion through interactions with leukocytes and the endothelium. Following antigen recognition, platelets often become activated. Through a diverse repertoire of mechanisms, activated platelets can directly sequester or kill pathogens, or facilitate pathogen clearance by activating macrophages and neutrophils, promoting neutrophil extracellular traps (NETs) formation, forming platelet aggregates and microthrombi. At times, however, platelet activation may also be injurious to the host, exacerbating inflammation and promoting endothelial damage and thrombosis. There are many gaps in our understandings of the role of platelets in infectious diseases. However, with the emergence of advanced technologies, our knowledge is increasing. In the current review, we mainly discuss these evolving roles of platelets under four different infectious pathogen infections, of which are dengue, malaria, *Esterichia coli* (*E. coli)* and *staphylococcus aureus S. aureus*, highlighting the complex interplay of these processes with hemostatic and thrombotic pathways.

## Introduction

Infectious diseases remain a leading cause of morbidity and mortality worldwide. Low platelet number, termed *thrombocytopenia*, is common in infectious diseases (also referred to at times as sepsis). The mechanisms are often multifactorial, but increased platelet clearance and/or decreased platelet production are common. Sepsis-associated thrombocytopenia has been recognized for many years and is a predictor of adverse outcomes.

Until more recently, the involvement of platelets in the pathogenesis of acute infectious diseases has been less often studied. Part of the reason might be the traditional dogma that platelets are primary effectors of hemostasis and thrombosis, rather than participating in host immune responses against infections. With the expansion of our knowledge in the last decade or so, it is increasingly recognized that hemostasis, thrombosis, and inflammation are tightly interconnected processes and that platelets are often the cell that bridge these three processes.

Classically, hemostasis is referred to as the process of clot formation under normal physiological situations to stop bleeding upon blood vessel damage (e.g., wound). Thrombosis is defined as clot formation under pathological situations (e.g., atherosclerosis plaque rupture, deep vein thrombosis, pulmonary embolism, and stroke – among other examples). Infections, particularly when acute, are known to increase the risk of thrombosis. For example, Kaplan et al. identified a cohort of septic patients, that approximately one-third of patients developed a deep vein thrombosis and/or pulmonary embolism (1–3). Although the reasons that infection trigger thrombosis are multifactorial and incompletely understood, inflammation is thought to be a contributing factor. In particular, there is increasing recognition that inflammation may directly activate the hemostatic systems, resulting in thrombosis (4, 5).

Inflammation is generally considered a process of various immune responses that causes clinical symptoms include heat, pain, redness, and swelling. Similar to thrombosis, inflammation can also be triggered by wounds, tissue damage, and pathogen invasion. The traditional separation of “thrombosis” and inflammation,” while helping to understand the key physiological processes upon injury or infection, may hinder an understanding of the complete picture. Moreover, pursuits of novel therapeutics too focused on one aspect of the disease may not be successful (6).

In recent years, words like “thromboinflammation,” “immunothrombosis,” and “immunohemostasis” have been used to describe responses/mechanisms that are involved in both thrombosis and inflammation (7–11). To our knowledge, “thromboinflammation” was initially used by Blair et al. in 2009 to describe their discovery that the activation of platelet toll-like receptor 2 (TLR2), a receptor best known for its role in triggering inflammation, also promotes thrombosis (7). In 2013, Engelmann used the word “Immunothrombosis” to describe thrombosis “triggered by” or “involved with” innate immune responses (8). While not of our invention, we and others have used “immunohemostasis” to describe hemostatic responses that involves immune players under physiologic situations, as compared to the pathologic property of “thrombosis” (12). To clarify our discussion below, here we will define “immunohemostasis” as an integrated process that includes the classic coagulation, clot formation, as well as the immune responses for pathogen trapping upon blood vessel damage without pathological consequences. In contrast, “thromboinflammation” will refer to pathological responses within the vasculature following blood vessel injury, invasion by a variety of pathogens, or non-infectious inflammatory triggers. We hope that by this definition, the process of thromboinflammation be an umbrella that considers thrombus formation, coagulation system activation, and innate and adaptive immunity as an integrated detrimental process. Thus, thromboinflammation is associated with diseases that are historically under separated categories. Some examples include thrombotic diseases like stroke, deep vein thrombosis, and myocardial infarction, infectious diseases such as bacteremia, viremia, and parasitemia, cancer metastasis through blood vessels, and disseminated intravascular coagulation (DIC).

In the past decade, efforts in better understanding the pathogenesis of infectious diseases have led to new discoveries of the critical roles that platelets have in thromboinflammation. In some settings, platelets may be protective through limiting pathogen dissemination, directly killing pathogens, and eliciting timely and adaptive host immune responses timely. In other situations, however, platelet responses during infection may be harmful. In our current review, we will focus on the interactions between platelets and classic immune cells during infectious diseases. We use dengue, malaria, *Esterichia coli* (*E. coli)* and *Staphylococcus aureus* (*S. aureus*) infections as specific pathogen examples to illustrate the thromboinflammation as an integrated process in which platelets actively participate. Platelets are also involved in many other infectious diseases outside the scope of this review. Readers are referred to several other excellent summaries of this topic (13–19).

## Platelets are Versatile Patrollers

Platelets are the smallest cells in blood circulation with a diameter of 2–3 μm under resting conditions (20). They are anucleate cells produced by fragmentation of the megakaryocyte extrusions into the vasculature, formed mostly in the bone marrow, although other sites of platelet genesis include the lung, spleen, and liver (21). Differentiated from hematopoietic stem cells or common myeloid progenitors (22–24), megakaryocytes and platelets share many myeloid lineage features, such as the expression of a panel of pattern recognition receptors (PRRs), phagocytosis of exogenous antigens, interactions with other immune cells, and the release of chemokines and cytokines upon activation (15, 20, 25, 26). Having a life span of 7–10 days in healthy humans and about 5 days in the mouse, it is estimated that about 100 billion platelets are produced every day in humans, with about 2 billion a day in mice (20). Platelets are mighty patrollers that constantly scan over the vascular endothelium and circulating leukocytes in a “touch and go” manner (27). Upon activation, platelets rapidly undergo massive plasma membrane extension (spreading), become activated, translocate and/or express multiple receptors on their surface that further enhance their aggregation with nearby platelets or leukocytes, or directly bind to and sequester extracellular pathogens (20). Platelets also degranulate and release pre-packed (or in some cases) newly-synthesized microbicide proteins and chemokines that facilitate pathogen destruction, signal immune cells, and promote inflammation (20). The reader is also referred to a recent review by Rossaint et al. that provides more detailed information on the platelet receptors and chemokines involved in inflammation (28).

Work from our group and others demonstrates that during pathogen invasion, platelets have alterations in their transcriptome and proteome that augment host defense mechanisms (29–31), although these changes may also result in adverse outcomes in some settings. These and other features make platelets effective and dynamic sentinels against bloodborne pathogens.

## Dengue Virus

**Figure 1:**
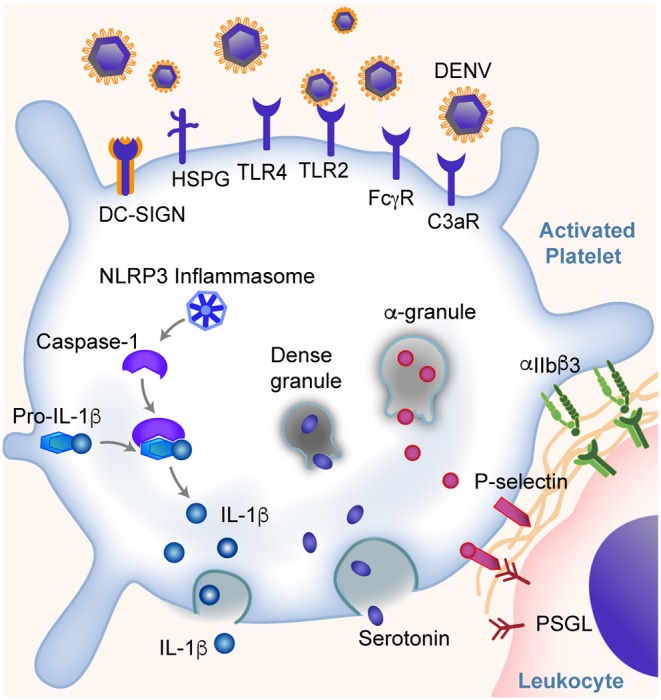
Platelets are key sentinel and effector cells during dengue infection. Platelets can directly bind dengue virus (DENV) and virus: IgG immune complexes through DC-SIGN and other receptors as listed. This binding leads to altered gene and protein expression in platelets and the activation of platelets. Some of these responses include the expression of IFITM3, assembly of NLRP3 inflammasomes, production and release of IL-1β, secretion of α-granule and dense granule contents, translocation of P-selectin to the platelet surface (allowing platelet-leukocyte interactions and signaling), and integrin α_IIb_β_3_ activation. Changes in platelets further trigger the platelet aggregation and thrombosis, endothelial inflammation and vascular leakage, and monocyte activation and cytokine production, and more. These responses span the classic pathogenesis of thrombosis and inflammation, and may contribute to hemorrhagic symptoms and shock in some patients.

Dengue virus (DENV) is a mosquito-borne, positive sense, single-stranded RNA virus of the Flaviviridae family, with five serotypes documented so far (32, 33). The annual incidence of dengue is estimated to be 390 million globally, with about 100 million individuals having clinically apparent symptoms (34). While in most individuals the disease is self-limited with a high fever as the only symptom, about 10% of patients have thrombocytopenia and hemorrhagic symptoms (termed dengue hemorrhagic fever, DHF). In severe cases of dengue infection, patients may develop dengue hemorrhagic shock (DHS) (35). Morbidity and mortality rates in these last two types of dengue infection can be rather high. Emerging and established studies highlight that platelets are implicated centrally in the pathogenesis of the disease. Figure 1 summarizes some of the responses of platelets during dengue infection.

Dengue virus can directly bind and activate human platelets via multiple receptors. The direct interaction was suggested from observations of dengue virus RNA and dengue-like particles in platelets from patients with dengue (36). Later studies demonstrated that the direct interactions between dengue and platelets are mediated through dendritic cell-specific intercellular adhesion molecule-3-grabbing non-integrin (DC-SIGN) and heparin sulfate proteoglycan receptors (HSPG) on platelets (37, 38). Intriguingly, the expression of DC-SIGN on platelets appears to decrease in some patients as dengue infection resolves (39). In addition, Chao et al. showed direct platelet activation by DENV nonstructural protein 1 (NS1) through interactions with TLR4 and TLR2 (40). Recently, Sung et al. showed DENV induces platelet activation via CLEC-2 (41). Following DENV binding, platelets become activated, undergo a series of changes that further amplify the thromboinflammation, which will be discussed later.

Similar to myeloid cells, platelets also express Fc receptors that are capable of binding IgG opsonized DENV complexes. This mediates, in part, the development of thrombocytopenia and DHF/DHS in patients infected with DENV. Fc receptors have recently been shown to play a central role in antibody-dependent enhancement (ADE) during dengue infection. ADE is the clinical setting whereby individuals previously exposed to (or immunized against) one dengue serotype have an increased risk of more severe dengue (e.g., DHF/DHS) when they become infected with a different dengue serotype (42, 43), suggesting an intimate relationship between antibody mediated inflammatory responses and platelets. The work by Katzelnick et al., which included a large cohort of more than 8,000 patients with dengue, reported that ADE and severity of dengue is associated with DENV-antibody titers between 1:21 and 1:80 (43). Moreover, during the early phase of DENV infection, the glycosylation of Fc regions and the ratio between IgG subclasses (IgG1/IgG2) appears to regulate platelet counts (42). Human platelets express FcγRIIA and FcγRIIIA receptors that can engage both IgG1 and IgG2 (31, 42, 44–46). Increased afucosylation (the absence of fucose on the Fc glycan) of the CH2 domain of the Fc region of both IgG1 and IgG2, together with an increased IgG1/IgG2 ratio, leading to altered binding affinity between IgG and FcγR receptors on platelets (42). In humanized FcR transgenic mice, this results in significant thrombocytopenia (42). Following binding of IgG immune complexes to platelets, platelets degranulate and release stored serotonin; this pathway is downstream of FcγRIIA signaling (47). In some settings, increased circulating levels of serotonin can contribute to systemic shock (47, 48).

Upon DENV infection, platelets can also be activated indirectly by complement C3, serotonin, or PAF. For example, platelets express complement receptor C3R (49, 50). Platelets from patients infected with dengue have increased IgG and C3 binding that is associated with increased platelet activation and clearance. This could not be completely blocked by FcγRIIA inhibition (51, 52). Moreover, platelets could be activated by serotonin via 5HT_2A_ receptors, which leads to increased platelet clearance and development of thrombocytopenia (53). Recently, Masri et al. showed that the serotonin is mainly synthesized in mast cells during DENV infection and subsequently internalized by platelets via 5HT_2_ receptors (53). Similarly, PAF receptor (PAFR) deficient mice also exhibited elevated platelet counts and decreased vascular permeability after DENV-II inoculation, as compared to wild type (WT) mice, suggesting that PAFR is also involved in the development of thrombocytopenia (54, 55). This further supports our notion that platelets have many myeloid cell features that mediate thromboinflammation.

Following activation of platelets during dengue infection, the global gene expression, proteomic, and lipodomic profilings of platelets are all altered to facilitate viral clearance (56–58). Unbiased next-generation RNA sequencing of platelets from patients and in megakaryocytes infected *in vitro* with dengue virus by recent work from Campbell et al. (56) suggests that megakaryocytes sense dengue infection and/or agonists generated during infection, and in response alter the repertoire of mRNAs invested into newly produced platelets (56). One of transcripts significantly upregulated in megakaryocytes and platelets during DENV infection was interferon-induced transmembrane protein 3 (IFITM3). IFITM3 protein was also increased in platelets from patients infected with DENV and cultured megakaryocytes exposed to DENV *in vitro*. IFITM3 in other cells restricts viral replication, therefore enhancing resistance to DENV infection (56). Interestingly, IFITM3 induction in human megakaryocytes not only reduced DENV infection of megakaryocytes but also reduced DENV infection in stem cells in the surrounding bone marrow niche. This study highlights the ability of megakaryocytes to participate effective in innate antiviral immune responses.

As mentioned above, through DC-SIGN and HSPG, TLR4, CLEC-2, and other receptors, DENV could activate platelets, cause thrombocytopenia and thromboinflammation in patients. Platelet activation is associated with the severity of thrombocytopenia and increased risk of DHF in patients with DENV (37, 51, 59, 60). A hallmark of platelet activation is the conformational change of integrin α_IIb_β_3_, which binds to fibrinogen and von Willebrand factor (vWF), triggering platelet adhesion to vascular endothelial cells and causing thrombosis (61). Activation of platelets also triggers degranulation and the release of a number of proteins including P-selectin, PAF, soluble CD40L (CD154), and serotonin—proteins that are often dichotomized into either pro-thrombotic or pro-inflammatory, but which could fall together under the term “thromboinflammation” (54, 55, 62, 63). P-selectin on platelets engage its ligand, P-selectin glycoprotein ligand (PSGL) on leukocytes, promoting proinflammatory cytokine production such as IL-1β and IL-8 by both platelets and monocytes (40, 62, 64). These cytokines induce enhanced permeability of endothelial cells and vascular leakage, and are associated with an increased risk of DHS in patients (64). P-selectin also tether platelets to PSGL on endothelial cells, and induces vascular endothelial damage (65, 66). Moreover, serotonin not only contributes to the development of thrombocytopenia. The stored serotonin could be released from a large number of platelets following platelet activation, lead to an increased concentration of serotonin in circulation, cause vasodilation and hypotension, and development of systemic shock (47, 48). Upon activation, platelets undergo apoptosis, with decreased mitochondrial potential, assembly of inflammasomes, increased phosphatidylserine (PS) exposure and P-selectin expression on the cell surface (37, 38, 67). This process further catalyzes thromboinflammation. For example, the apoptosis of platelets not only increases the phagocytosis of platelets by monocytes, but also triggers activation of the coagulation system, generation of thrombin, and formation of thrombi (68–70). In addition, during DENV infection, the release of angiopoetin-1 by platelets is reduced. This is associated with dampened inhibition of angiopoetin-2, and increased endothelial damage in patients with DHS (71).

Dengue activated platelets are associated with increased assembly of inflammasome NLRP3, which activates caspase-1 and triggers apoptosis within platelets, and promotes platelet aggregation (67, 72). This leads to a reduced platelet lifespan and contributes to the development of thrombocytopenia in dengue patients (63, 67). The NLRP3 inflammasome assembly in platelets also leads to the synthesis of IL-1β by platelets and its subsequent secretion into plasma and packaging into microvesicles (67). Platelet activation and apoptosis is higher in patients with DHF than without DHF, and correlates with *in vitro* phagocytosis of platelets by macrophages through a phosphatidylserine-recognizing pathway (37, 51). Furthermore, NLRP3 and FcγRIIIA have been shown to induce dengue-triggered hemorrhage in mice synergistically (73). NLRP3 has also been correlated with increased low-density lipoproteins (LDL) and decreased high-density lipoproteins (HDL), suggesting extravascular effects to host lipid metabolism following NLRP3 activation (74). However, the role of platelets in this pathological process remains unclear.

In addition to antibody-dependent platelet activation, platelets may also engage antigen specific T cells by presenting dengue antigens through MHC class I. For example, a proteomic study by Trugilho et al. revealed proteasome subunit proteins and HLA class I antigen presentation pathway proteins as the most significantly upregulated in platelets during dengue (57). In addition, platelets express several T cell co-signaling ligands, such as CD40, CD86, ICOSL, and upon activation, are capable of cross-presenting exogenous antigens *in vitro* and stimulating antigen specific T cell responses (75). It seems possible that following direct binding of virus or through FcγRIIA, platelets internalize dengue virus, degrade dengue antigens in their immunoproteasomes, and present antigen peptides through HLA class I for recognition by CD8^+^ T cells.

Platelets are also involved in other pathological mechanisms of dengue infection. For example, platelets may increase endothelial barrier permeability due to decreased S1P levels, or promote DENV replication in monocytes through the release of platelet factor 4 (PF4, also known as CXCL4) (64, 76–78). Platelet-derived microparticles may also play a role in the pathogenesis of the disease, as they are notably increased in thrombocytopenic patients without bleeding, and decreased in thrombocytopenic patients with bleeding (67). A summary of some of these identified mechanisms can be found in Figure 1.

## Malaria

Malaria, also a mosquito-borne infectious disease, is caused by systemic infection with parasites of the *Plasmodium* group. In humans, five species of *Plasmodium* have been identified that cause malaria: *P. falciparum, P. malariae, P. ovale, P. vivax*, and *P. knowlesi*. Of these, *P. falciparum* is the most common (79). Malaria has been a major health concern worldwide for decades, with an estimated incidence of more than 200 million cases a year (79). The parasitic sporozoites are transmitted from the mosquito's saliva into human bloodstream, and subsequently infect hepatocytes where they mature into schizonts. Eventually, the infected hepatocytes rupture, releasing merozoites into the bloodstream. *Plasmodium* then invade erythrocytes, replicating until the cells burst. This cycle is repeated, typically causing fever each time the erythrocytes burst.

Common characteristics of malaria include headache, cyclical fevers, anemia, and thrombocytopenia. In the study by de Mast et al. healthy volunteers were infected with *P. falciparum* and developed thrombocytopenia at the earliest phase of blood-stage infection (80). Thrombocytopenia is so common in patients that platelet count has been proposed as an affordable and fast diagnostic test for malaria in low-income regions, with a reported sensitivity of 60% and specificity of 88% (81–83). Recently, Gardinassi et al. integrated plasma metabolomic data and whole blood transcriptomic data obtained from volunteers infected with *P. vivax* (84). They found that platelet activation together with changes in IFN signature modules and T cell signaling are the top three most significantly changed processes (84).

**Figure 2:**
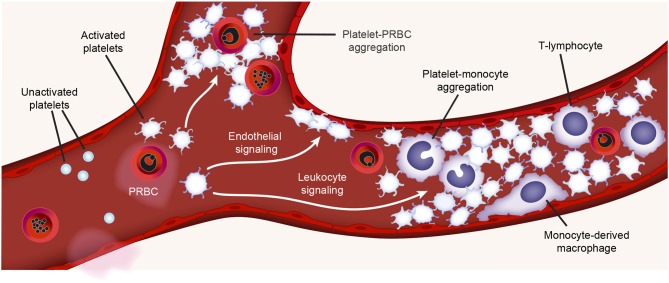
Platelet activities during malaria infection. During malaria infection, platelets may be found in the vasculature closely surrounding plasmodium-infected RBCs (PRBCs) or interacting with monocytes (platelet-monocyte aggregates) or lymphocytes. Toxins generated during malaria infection (e.g., hemozoin) may active platelets. Platelet interactions with monocytes trigger pro-inflammatory cytokine synthesis by monocytes. Figure is adapted from Rondina et al. (94) with permission obtained from Elsevier.

A major cause of morbidity and mortality in patients with malaria is cerebral malaria (CM) (85, 86). Increased platelet accumulation has been documented in the brain microvasculature of children who died of CM, as well as in animals with experimental cerebral malaria (ECM) (85–89). These platelets were often found aggregated with Plasmodium parasites or leukocytes, together with increased vWF staining (18, 90–92). Platelet activation and thrombosis may precede leukocyte infiltration (88). Moreover, recent proteomic studies performed on postmortem brain tissue obtained from CM patients revealed that proteins involved in platelet activation and coagulation were upregulated (93). Thus, platelets and leukocytes (as well as platelet-leukocyte aggregates) are associated with CM. Figure 2 highlights a schematic representation of some of the interactions between activated platelets and other circulating blood cells during malaria infection.

Both platelets and platelet derived microparticles (PMPs) can directly bind *Plasmodium* infected RBCs (PRBCs) (88, 89, 95, 96). This binding is mainly mediated by CD31 (PECAM-1) and CD36 (GPIV) on platelets and the *P. falciparum* erythrocyte membrane protein 1 variant on PRBCs (89, 95, 96). After binding, platelets can directly kill the intraerythrocytic Plasmodium *in vitro* (97, 98). In patients, platelets can directly kill all major malaria parasites *in vivo*, and the decreased platelet count has partially been attributed to the increased binding to PRBCs (99). In some—but not all—studies, the severity of thrombocytopenia has been associated with increased parasitic density and adverse outcomes (98, 100, 101). The significance of platelets in parasite clearance remains somewhat uncertain, as the physiological platelet to RBC ratio ranges from ~1:10 to 1:50, and platelet-bound PRBCs have been found in less than 5% of the total PRBCs in patients infected with malaria (98, 99, 102). Nevertheless, considering that about 100 billion platelets are produced daily (for comparison, about 200 billion RBCs are produced daily), and platelet-mediated parasite killing could be occur very rapidly (e.g., minutes), it remains plausible that platelets contribute to parasitic killing in humans, directly or indirectly. This remains an area of active investigation in the field.

One chemokine implicated in platelet-mediated parasite clearance is PF4. Following direct platelet-PRBC binding, platelets release PF4 which engage the Duffy antigen receptor (Df) on PRBCs. This engagement induces disruption of the parasite digestive vacuoles and terminal deoxynucleotidyl transferase deoxyuridine triphosphate nick-end labeling of parasite nuclei (TUNEL+), without lysing PRBCs (99, 103). Following this initial discovery, subsequent studies have shown that PF4-driven antiparasitic activity is mainly mediated by the C-terminus of PF4. A synthetic PF4 derived cyclic peptide, named cPF4PD, showed promising killing of parasites in PRBCs without lysing normal RBCs (104, 105). Recent work by Wang et al. showed that the host can upregulate PF4 production in malaria by activating the transcription factor E74 like ETS transcription factor 4 (ELF4) in megakaryocytes (106).

However, the PF4 released from platelets is not entirely protective, as in some settings PF4 release may trigger inflammation. For example, in a mouse model of experimental cerebral malaria (ECM), platelet accumulation and PF4 release in the brain led to significantly increased blood brain barrier permeability and T cell infiltration. In these models, PF4 deficient mice have decreased T cell infiltration and were protected from the development of ECM (89). Further studies showed that platelet-derived PF4 activated the transcription factor KLF4 in monocytes and promoted monocytes to produce proinflammatory cytokines such as IL-6 and TNFα, which are important in the pathogenesis of ECM (88).

Platelets also have been shown to participate in thromboinflammation during malaria infection through other mechanisms. For example, platelets are the major source of IL-1β, which protects against ECM development in experimental models of malaria (107). Additionally, plasma vWF levels (including ultra-large vWF multimers) are increased in patients with malaria (18, 92, 108, 109), also associated with increased GPIb shedding from platelets and the development of thrombocytopenia (110). The readers are referred to several excellent reviews on this topic (18, 81, 111).

## Gram-Negative Bacterial Infections

Bacteria are commonly classified into Gram-positive and Gram-negative species based on their cell wall structures. Gram-positive bacteria feature a thick layer of peptidoglycan, which is evident by Gram staining. Gram-negative bacteria have a thin layer of peptidoglycan covered by a layer of outer membrane and lipopolysaccharides (LPS). Infection by pathogenic bacteria can cause thromboinflammation locally or systemically, such as via endothelium barrier damage and lung edema, intestine inflammation and diarrhea, platelet activation, and thrombocytopenia, coagulation activation and disseminated intravascular coagulation (DIC). In experimental models of infection, depleting platelets prior to infection often leads to increased mortality, suggesting an important role of platelets in host survival during bacterial infections (112–116).

The Gram-negative bacterium *E. coli* is a major cause of urinary tract infections. Strains of *E. coli*, as well as LPS purified from *E. coli* are widely used in research and these tools have improved our understanding of many host immune responses in platelets and other cells. We and others have shown direct interactions between platelets and *E. coli in vitro* (117). Direct interactions between platelets and *E. coli* have been captured *in vivo* recently using super-resolution microscopy (118). These investigators captured real-time, *in vivo* platelet migration toward *E. coli* in mouse liver sinusoids within hours after infection, as well as *in vitro* platelet migration toward and bundling of *E. coli* bacteria within minutes (118).

Human platelets express toll-like receptors (TLRs) 1–10 (at the mRNA and/or protein level), and mouse platelets express TLRs 1-8 (31, 119–121). Of these, TLR4, an LPS specific receptor, was the first TLR well characterized on platelets (122). In macrophages and dendritic cells, LPS activates the TLR4/MyD88 signaling, triggers inflammation (123, 124). In platelets, LPS activates the TLR4/MyD88 and signals downstream via cGMP/PKG-dependent pathway (125). Administration of LPS to rats resulted in platelet activation and adhesion to the endothelium through P-selectin and integrin GPIb receptors and the development of thrombocytopenia in rats within 30 min (122). Subsequently, Cognasse, Andoneigui and Aslam et al. showed independently that both human and mouse platelets have functional TLR4 on their surface (113, 121, 126).

Upon LPS stimulation and TLR4 activation, a number of events happen to platelets that cause thromboinflammation (127). LPS stimulation signals to human platelets to process tissue factor (TF) pre-mRNA into mature mRNA, with subsequent increases in TF protein, which is procoagulant (117, 128). This is a unique bacteria stimulated platelet response that has been not reported in any other infectious diseases. LPS also induces some other changes in platelets as we have mentioned above. Similar like activation during DENV infection, platelets can also be activated and undergo apoptosis upon LPS stimulation (129), and triggers the secretion of thrombo-inflammatory factors, including P-selectin at low LPS concentrations and CD40L, TNF-α, and PF4 at higher LPS concentrations (127, 130, 131). From an evolutionary perspective, this might serve as an adaptive rheostat to the host, perhaps by limiting the scale of thromboinflammation when the bacterial load is low (125, 126, 128, 131). Moreover, LPS stimulates platelets to opsonize IgG immune complexes and enhances the phagocytosis of platelets by macrophages, suggesting there may be a synergistic effect of TLR4 and FcR on platelets (132). LPS also stimulates platelets to interact with neutrophils through the interactions between P-selectin, PSGL-1, and lymphocyte function-associated antigen 1 (LFA-1) (112, 114, 133). Neutrophils reciprocally trigger platelet activation and thromboxane generation during *E. coli* infection (114). In addition, upon LPS stimulation, platelets maintain vascular integrity and prevent leukocyte infiltration via CLEC-2 (116).

**Figure 3:**
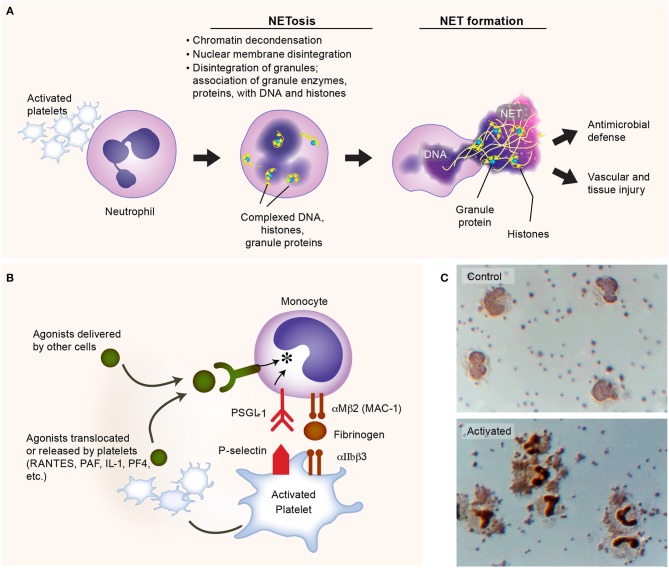
Activated platelets induce formation of neutrophil extracellular traps (NETs). **(A)** Platelets are commonly activated during Gram-negative bacteria infections, and express multiple receptors that could facilitate the process of NET formation (NETosis), such as P-selectin_._ The lattices of chromatin, histones, and granule enzymes (NETs) play a critical role in pathogen clearance and may also induce thromboinflammatory responses, potentially contributing to vascular and tissue injury. **(B)** Activated platelets interact with monocytes, inducing the synthesis of inflammatory mediators. **(C)** Upper panel: isolated human platelets and monocytes incubated under control conditions. Lower panel: formation of platelet-monocyte aggregates and nuclear translocation of nuclear factor kappa B (NF-κB) in monocytes when the platelets were activated with nanomolar concentrations of thrombin. Figure is adapted from Rondina et al. (94) with permission obtained from Elsevier.

Upon Gram-negative bacterial infections, platelets are actively involved in the generation of neutrophil extracellular traps (NETs) (112). NETs are extracellular lattices of chromatin, histones, and granule enzymes extruded by neutrophils upon activation (Figure 3A), via a unique process termed “NETosis” (134). NET formation is an efficient mechanism for trapping both Gram-positive and Gram-negative bacteria. Platelets mediate key aspects of NET formation. Although neutrophils can form NETs without platelets when infected with Gam-positive or Gram-negative bacteria, platelets are able to significantly expand the surface area of NETs *in vitro* (112). *In vivo* depletion of neutrophils or platelets impairs NET formation and significantly impedes bacterial clearance (112, 135).

Multiple surface receptors on platelets and secreted factors by platelets regulate NET formation. For instance, platelet-derived P selectin plays an important role in the early stage of NETosis, facilitating platelet-neutrophil direct interactions (114, 136). Platelet GPIbα, a well-studied receptor in hemostasis and thrombosis, has been found recently triggers the activation of neutrophils and extracellular vesicle release from neutrophils (114). In addition, another important receptor in hemostasis and thrombosis, the integrin αIIbβ3 on platelets, this also mediates NETosis, as the deficiency of integrin αIIbβ3 impairs NETs formation (135). Although NETs trap bacteria efficiently, exaggerated or misplaced NET formation may also be deleterious. Histones, serine proteases, and cathepsin G released by activated neutrophils can activate platelets, promoting coagulation, endothelial damage, and thrombosis (133, 137–139). Understanding the roles of NET formation in adaptive and maladaptive host responses remains an active area of investigation. We refer the reader to the recent review article by Zucoloto et al. for more detailed discussions on the platelet-neutrophil interactions that illustrate the intimate relationship between inflammation and thrombosis (19, 140).

Another example of the versatility of platelets and thromboinflammation as an integrated process is the synergistic signaling of FcγRIIA receptors and platelet integrin αIIbβ3 (a canonical hemostatic receptor) upon *E. coli* infection. In addition to activation through TLR4, platelets could also be activated by *E. coli* via FcγRIIA receptors, provided simultaneous integrin αIIbβ3 signaling (45). Either absence of IgG or blockage of αIIbβ3 signaling would abolish the aggregation of platelets when incubated with *E. coli* (45, 141). Recent work by Palankar et al. demonstrates that human platelets directly kill *E. coli* in mechanisms that require FcγRIIA and PF4 (142). As with malaria, PF4 is also central to effective *E. coli* killing. Disruption of platelet cytoskeletal functions also reduced the efficacy of *E. coli* killing by platelets. Moreover, the complement C3 opsonization of E. coli facilitates the formation of platelet-bacteria aggregates which is important in the induction of adaptive immune responses (143). Together, these findings suggest that platelets accumulate on bacteria, releasing antimicrobial α-granule contents that effectively kill *E. coli* (142). While not a central focus of this review, platelets interact with other leukocytes, including monocytes, forming stable platelet-leukocyte aggregates that promote the release of agonists from platelets and subsequent pro-inflammatory cytokine synthesis by monocytes (Figures 3B,C). Interestingly, in aging (where the risk of infection rises substantially), these interactions may be upregulated—potentially contributing to cytokine release injurious to the host (144). Whether these aging-dependent responses contribute to adverse clinical outcomes in sepsis remains an area of active investigation.

Bacteria also possess endogenous mechanisms to counteract host defenses. Some of these directly affect human platelet activities. As one example, work from our group demonstrated that pathogenic *E. coli* bacteria isolated from infected patients induced platelet apoptosis via calpain-mediated degradation of the cell survival protein Bcl-x_L_ (145). This was accompanied by impaired mitochondrial membrane potential and lateral condensation of actin. Degradation of Bcl-x_L_ was driven by alpha hemolysin, a pore-forming toxin produced by *E. coli*. Interestingly, clinical isolates of *S. aureus* (a Gram-positive bacteria discussed below) that produced alpha toxin (α toxin, also known as α hemolysin) degraded in Bcl-x_L_ platelets (145). These findings suggest a mechanism whereby bacterial pathogens contribute to thrombocytopenia.

## Gram-Positive Bacterial Infections

Gram-positive bacterial infections, especially multidrug resistant pathogens, are a major global health challenge (146). *S. aureus, Listeria monocytogenes (L. monocytogenes)*, and *Streptococcus pneumoniae (S. pneumoniae, or pneumococcus)* are common pathogens causing substantial morbidity and mortality. Here we highlight the mechanisms of platelet-mediated thromboinflammation during *S. aureus* infections, but readers are referred to the recent review by Anderson and Feldman on platelets in pneumonia and other excellent reviews as we have mentioned above (13, 14, 147).

**Figure 4:**
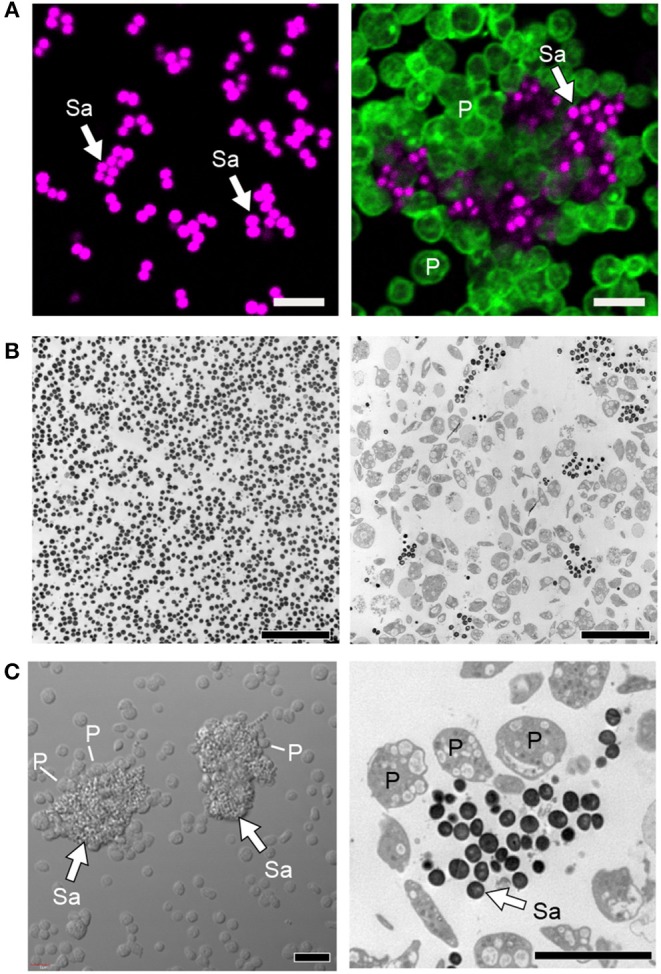
Platelets sequester *S. aureus* and promote thromboinflammation. **(A)** Confocal and **(B)** transmission electron microscopy of cultured staphylococcus *aureus* (Sa) incubated in the presence (right panels) or absence of platelets (P, time = 240 min). **(C)** Differential interference contrast (left panel) and transmission electron (right panel) microscopy of clusters of *S. aureus* (Sa, white arrows) surrounded by platelets (P). Scale bars = 5 μm. After sequestration of *S. aureus*, platelets become activated, form aggregates *in vitro* and microthrombosis *in vivo*, interact with macrophages and neutrophils, trigger NETs formation, and also shuttle bacteria to splenic dendritic cells to activate CD8^+^ T cell responses. Figure adapted from Kraemer et al. (151) with permission.

For decades, *S. aureus*, including methicillin sensitive and resistant *S. aureus*, i.e., MSSA and MRSA, was known to interact with, and activate platelets. In 1964, Siegal et al. showed that a toxin from staphylococcal bacteria could induce platelet morphological changes observed by electron microscopy images and inhibition of platelet rich plasma clotting (148). In the 1970s, Clawson and White showed that among several strains of bacteria able to directly bind platelets and induce platelet aggregation and adhesion, *S. aureus* was the most potent (149, 150). Interestingly, at low bacterial numbers, platelets bind to bacteria without forming substantial platelet-platelet aggregates, suggesting there may be favored interactions between bacteria and platelets under these conditions. At higher bacterial numbers, platelet aggregation is induced and bacteria can be found encased in platelet-platelet aggregates (Figure 4) (149, 150). More recently, Wong et al. showed that upon MRSA infection *in vivo*, platelets scan liver Kupffer cells and rapidly (within minutes of infection) recognize MRSA on the surface of Kupffer cells (27). This triggers aggregation within sinusoids that limits bacterial spreading (27). Additionally, platelet depleted mice have significantly increased mortality upon MRSA infection *in vivo* (27).

There is heterogeneity for clinically isolated *S. aureus* species from patients to bind platelets (27, 152). When *S. aureus* binds to platelets, it appears to be in a saturable and reversible manner, suggesting there are receptor-ligand mediated interactions between platelets and *S. aureus* (153). Recently, platelets are shown to be capable of spreading and enclosing bacteria *in vitro* (118, 151).

Platelets can directly bind *S. aureus* antigens through multiple receptors. For example, activated human platelets express gC1qR, which could directly bind *S. aureus* protein A without complement opsonization (154). In addition, platelets could be directly activated by the α toxin, the Staphylococcal superantigen-like 5 (SSL5), extracellular adherence protein (Eap), chemotaxis inhibitory protein of S. aureus (CHIPS), the formyl peptide receptor-like 1 inhibitory protein (FLIPr) and other proteins of *S. aureus* (151, 155–158). Alpha toxin is the major cytotoxic virulent factor of *S. aureus*, capable of forming heptamers and develop pores on target cell membrane, causing cell death. Incubation of α toxin with washed platelets induced morphological changes of platelets in suspension, including visibly lysed shape, smaller in size, and decreased content of intracellular granules (159). The incubation of α toxin also impaired the spreading capability of platelets on collagen and fibrinogen (159). In addition, α toxin can form complex with ADAM10 on platelets, a widely expressed zinc-dependent metalloprotease, and activate the latter (160). The activated ADAM10 then proteolyze GPVI on platelets, and impede platelet adhesion to collagen, and *in vivo* reduce platelets activation and accumulation at sites of infection (155, 161). These mechanisms facilitate bacteria evasion and spreading. Furthermore, *in vivo*, the α toxin induced platelet aggregation in the liver and kidney and associated organ failure has been shown by Surewaard et al. (162). In contrast, platelets also evolved with protective responses following α-toxin encounters. For example, α toxin induces the release of multiple microbicidal proteins from platelets, such as thrombin-induced platelet microbicidal protein-1 (tPMP-1) and human β defensin-1, which significantly suppress bacterial growth and trigger NETs formation (151, 152). Alpha toxin also triggers integrin αIIbβ3 dependent platelet aggregation and increased protein synthesis of Bcl3, which promotes clot retraction, stabilizes thrombus within the vasculature (159). In addition, α toxin can indirectly bind platelets when opsonized by complement C3b, which promotes platelet aggregates as well as platelet-macrophage and platelet-neutrophil interactions (161, 162). These platelet-monocyte interactions can induce NLRP3 inflammasome and IL-1β production in monocytes; this may happen in platelets but as of yet remains unproven (163–165). SSL5 is a member of the SSL-family proteins that has been shown evolved in *S. aureus* evasion. It can directly bind either GPVI or GPIbα on platelets and signal downstream, induce activation of integrin αIIbβ3 and P selectin on platelets (157). Activated αIIbβ3 further promotes platelet aggregation, platelet-leukocyte aggregates, and adhesion to endothelial cells. Increased P-selectin also binds SSL5, as well as monocytes and neutrophils (156). In addition to direct binding, platelets can rapidly (within ~1 min of infection) bind complement opsonized bacteria, including *S. aureus, in vivo* (166). This may play a critical role in shuttling bacteria to the splenic DCs to trigger host CD8^+^ T cell responses (143, 166).

## Summary and Perspectives

Small in size and abundant in number, platelets are effective sentinels constantly roaming the vasculature, quickly sensing and responding to invading pathogens. Many of the responses by platelets to pathogen invasion bridges hemostatic, inflammatory, and immune continuums: the activation of platelets leads to the expression of activated integrin α_IIb_β_3_ and P-selection on the plasma membrane, formation of platelet aggregates and thrombosis, adherence and damage to the endothelium, increased interactions with macrophages and neutrophils, promotion of NETs formation, release of cytokines. These bridging features contributed to the evolution of the concept of thromboinflammation. Undoubtedly, the field will continue to see new discoveries expanding the armamentarium of platelet functions during infectious diseases. Exciting technological innovations are likely to continue to facilitate many of these discoveries. Sequencing techniques, such as next-generation RNA-sequencing and ribosomal footprint profiling, have already uncovered important new insights into the rich and dynamic nature of the platelet transcriptome and proteome (31, 167–170). Moreover, efforts to integrate sequencing data with machine-learning strategies may uncover new insights (84, 170). Proteomic studies of platelet lysates and granules have provided another layer of unbiased information about numerous proteins released by platelets and/or stored in their granules. Many of these include proteins synthesized within platelets, packaged from megakaryocytes, or internalized from the extracellular environment (84, 171–173). The application of super-resolution microscopy, including single-molecule localization microscopy (SMLM) and structured illumination microscopy (SIM), will provide unparalleled opportunities to visualize platelet granules and gather information about intracellular protein localization (174–176). The incredible evolution of our understanding of anucleate platelets in functions beyond just hemostasis has been an exciting journey and further discoveries will likely continue to expand the role of platelets in infectious diseases.

## Author Contributions

LG and MR designed and wrote the paper. Both authors reviewed and critically edited the manuscript.

### Conflict of Interest Statement

The authors declare that the research was conducted in the absence of any commercial or financial relationships that could be construed as a potential conflict of interest.
